# Effect of different sources of dietary protein on muscle hypertrophy in functionally overloaded mice

**DOI:** 10.1016/j.bbrep.2019.100686

**Published:** 2019-09-10

**Authors:** Shinya Aoyama, Rina Hirooka, Takeru Shimoda, Shigenobu Shibata

**Affiliations:** a Laboratory of Physiology and Pharmacology, School of Advanced Science and Engineering, Waseda University, Tokyo, Japan; b Organization for University Research Initiatives, Waseda University, Tokyo, Japan

**Keywords:** Protein source, Muscle hypertrophy, Milk protein, Plant protein, BW, body weight, BCAA, branched-chain amino acid, CAS, caseinate, epi, epididymal, FSR, fractional synthetic rate, ret, retroperitoneal and perirenal, SOY, isolated soy protein, WHE, whey protein concentrate

## Abstract

Dietary protein intake is important for skeletal muscle protein synthesis. In this study, we investigated the differential effect of protein sources on hypertrophy of plantaris muscle induced by surgical ablation of gastrocnemius and soleus muscles. Six-week old mice were fed diets containing caseinate, whey, or soy as protein sources for 2 weeks. Plantaris muscle hypertrophy was induced by a unilateral ablation of synergistic muscles after a week. Food intake of soy protein-fed mice was higher than that of caseinate and whey-fed mice, resulting in higher body and fat weights. Plantaris muscle weight in sham-operated mice was not different across the groups. Overload-operated plantaris muscle weight and increased ratio of overloaded muscle to sham-operated muscle weights were higher in caseinate-fed mice than in whey- and soy protein-fed mice, suggesting caseinate as a promising protein source for muscle hypertrophy.

## Highlights

• Type of dietary protein source affects functionally overloaded muscle hypertrophy.
• Caseinate intake induces higher muscle hypertrophy compared to whey and soy protein.
• Dietary protein sources have no effect on non-overloaded muscle weight.
• There is no effect of dietary protein sources on locomotor activity.

## Graphical abstract

**Figure:**
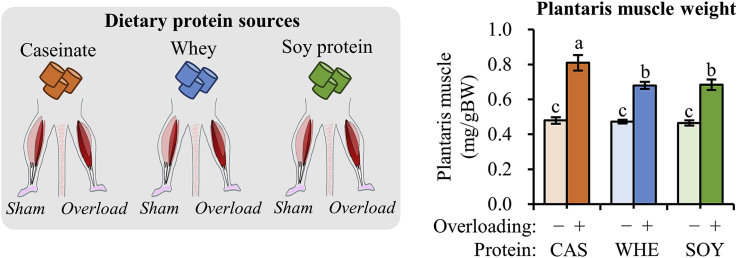

## 1 Introduction

Skeletal muscles play important roles in extending healthy life expectancy. Reduction of muscle mass and strength with aging is considered to be one of the major causes of disability in older people [1]. For example, handgrip strength is related to cognitive function, as reported from a 7-year study [2], and muscle strength during midlife has been reported to predict the possibility of functional limitations and disability 25 years later [3]. Dairy protein intake is negatively-related to the age-related loss of lean mass [4], and combination of protein supplementation and resistance exercise has been shown to result in higher muscle mass and strength compared to exercise alone [5]. Therefore, protein intake is important for muscle hypertrophy and health, although comparative study of protein sources still remain insufficient.

Dietary proteins are obtained from various food sources such as meat, milk, and vegetables. Caseinate, whey, and soy protein are not only common protein sources, but also provide high-quality protein [6]. Several clinical studies have revealed differential physiological responses to protein ingestion based on protein sources [[7], [8], [9], [10]]. The transient increase of blood amino acid level due to whey protein intake is faster than that due to caseinate intake, suggesting the digestion and absorption of whey protein to be relatively easier [[7], [8], [9], [10]]. Blood absorption rate of soy protein is reported to be between that of whey and caseinate [11].

For muscle protein synthesis after exercise, in humans, fractional synthetic rate (FSR) is higher after whey protein ingestion than after caseinate and soy protein ingestion [11], although 1–6-h FSR after heavy resistance exercise and ingestion of whey protein is similar to that in case of caseinate ingestion [10]. Dietary whey protein has been reported to be more capable of supporting muscle protein synthesis than soy protein in a clinical study [12]. Similar responses of FSR to protein ingestion were also observed in animal studies. Kanda et al. had reported that FSR, after exercise and administration of whey protein, is similar to that after caseinate administration, and seems to be higher than that after soy protein administration, although statistical analysis could not be performed [13]. Whey protein caused a significantly higher FSR compared to caseinate and soy protein at 60 min after feeding, and caseinate caused a significantly higher FSR compared to soy protein at 120 min after feeding [13]. Peak FSR, under no-exercise condition, is also reported to be different between whey protein and soy protein (45 min and 90 min, respectively) [14]. These reports convey the effects of intake of a single intake of protein source. However, in case of long-term intake, effect of different protein sources on muscle mass remains uncertain.

Muscle protein is maintained by a balance between synthesis and breakdown. When synthesis is higher than degradation, muscle hypertrophy is induced. Therefore, exercise and subsequent protein ingestion are important for muscle hypertrophy. Surgical ablation of gastrocnemius and soleus muscles was used to generate a model of resistance training-induced muscle hypertrophy of plantaris in mice [15]. In this study, we used this model as an evaluation of muscle hypertrophy due to protein ingestion. We examined the chronic effect of daily protein sources, caseinate, whey, and soy protein, on muscle hypertrophy in a resistance training-mimicking model.

## 2 Material and methods

### 2.1 Animals and experimental design

Male Kwl:ICR mice (5-week old, *n* = 32) were obtained from Tokyo Laboratory Animal Science (Tokyo, Japan). Animals were maintained in a conventional room (temperature: 22 ± 2 °C, humidity: 60 ± 5%, 12-h:12-h light-dark cycle; lights on at 8:00 a.m.) with access to food and water *ad libitum* (AIN-93G, Oriental Yeast Co., Tokyo, Japan). After 7-day acclimatization to the laboratory conditions, the mice were randomly divided into three groups, assigned to normal diet containing 17.7 (g%) protein as either calcium caseinate (CAS), whey protein concentrate (WHE), or isolated soy protein (SOY) (n = 11, 11, and 10, respectively) for a total of 2 weeks. After feeding on the switched food for 7 days, ablation of the synergistic gastrocnemius and soleus muscles was performed; another 7 days later, the mice were sacrificed and their plantaris muscle, liver, kidney, spleen, and fat (epididymal, retroperitoneal, and perirenal) were harvested and weighed. Body weight and food intake in each group were measured once a week. Experiments were approved by the Committee for Animal Experimentation at Waseda University (approval number: 2018-A019) and all animals were treated in accordance with the committee's guidelines.

### 2.2 Protein supplementation

Calcium caseinate, whey protein concentrate, and isolated soy protein were purchased as commercial products. Protein content of each protein was measured by the Kjeldahl method [16]. Crude protein = % N × 6.38 for CAS and WHE, and × 5.71 for SOY [17]. Nutritional composition of diets and amino acid composition of each protein are shown in Table 1, Table 2, and Supplemental Table 1.

**Table 1:** Nutritional composition of diets.

(g/100 g diet)	CAS	WHE	SOY
Nitrogen free extract	57.6	56.9	56.5
Protein	18.1	18.1	18.1
Fat	7.6	8.4	8.5
Ash	3.7	3.4	4.0
Moisture	8.1	8.3	8.0
Fibre	5.0	5.0	5.0
Energy (kcal/100 g diet)	371.3	375.0	374.4

**Table 2:** Amino acid composition (g/100 g diet).

	CAS	WHE	SOY
	g/100 g diet		
Ala	0.56	0.97	0.91
Arg	0.69	0.49	1.51
Asp	1.25	2.01	2.34
Cys	0.07	0.47	0.23
Glu	4.04	3.28	3.85
Gly	0.33	0.34	0.83
His	0.69	0.45	0.67
Ile	0.94	1.20	0.93
Leu	1.74	2.04	1.60
Lys	1.16	1.41	1.31
Met	0.51	0.36	0.24
Phe	0.94	0.61	1.05
Pro	2.16	1.18	1.08
Ser	1.07	0.97	1.07
Thr	0.81	1.38	0.81
Typ	0.23	0.37	0.26
Tyr	1.06	0.59	0.78
Val	1.11	1.04	0.91
Total	19.38	19.16	20.37

CAS,caseinate; WHE, whey protein concentrate; SOY, isolated soy protein.

### 2.3 Surgical procedure

In order to initiate overload-induced hypertrophy of plantaris muscle, unilateral ablation of the synergistic gastrocnemius and soleus muscles was performed, as described previously [18,19]. Incision in the contralateral leg was closed without surgical ablation of distal tendons, and its plantaris muscle served as a sham muscle. The animals were sacrificed 1 week after the surgery, and plantaris muscles of both hind limbs were excised and weighed. The hypertrophy ratio of plantaris was calculated from the ratio of overload-induced plantaris muscle weight per body weight to sham plantaris muscle weight per body weight.

### 2.4 Locomotor activity analysis

Locomotor activity of selected mice (n = 5–6) was continuously monitored with an infrared sensor (F5B; Omron, Kyoto, Japan) and analyzed with ClockLab software (Actimetrics, Wilmette, IL, USA) as previously described [20].

### 2.5 Statistical analysis

All data are expressed as mean ± standard error of the mean (SEM). Statistical analysis was performed using the SPSS software (version 25). Data were confirmed for normal distribution and homoscedasticity using Kolmogorov-Smirnov test and Levene's test, respectively. After the confirmation, statistical significance was determined by one-way ANOVA with a Tukey test. A *P*-value of <0.05 was considered to indicate a statistically significant difference.

## 3 Results

### 3.1 Body weight and food intake

**Fig. 1:**
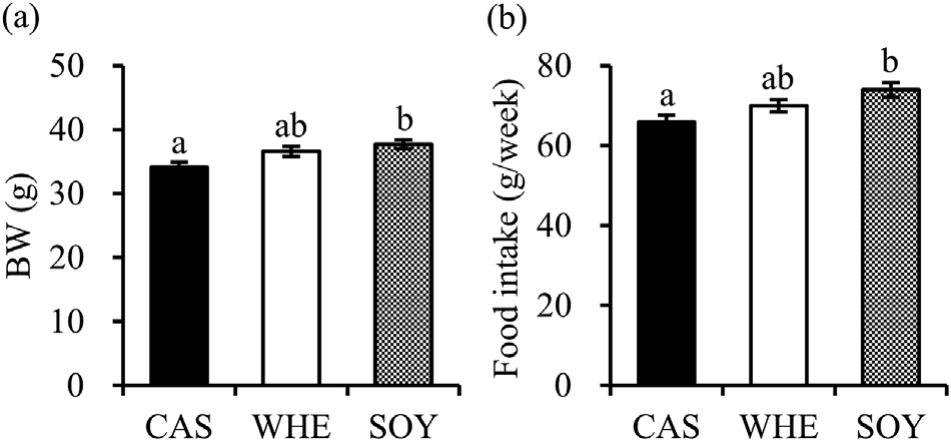
Effects of protein source on body weight and food intake. (a) Body weight (b) Daily food intake. Different protein source (caseinate; CAS, whey; WHE, soy; SOY) were administered for 2 weeks. The values are expressed as means ± SEM (n = 10–11). Means without a common letter are significantly different (*p* < 0.05), as per one-way ANOVA and Tukey test for multiple comparisons.

Change in body weight during the experiment is shown in Fig. 1. Body weight increased with time due to overall growth. Body weights in SOY group, after 2 weeks, were statistically higher than in CAS group. Consistent with body weight gain, average food intake of SOY was also higher than of CAS.

### 3.2 Muscle weight

**Fig. 2:**
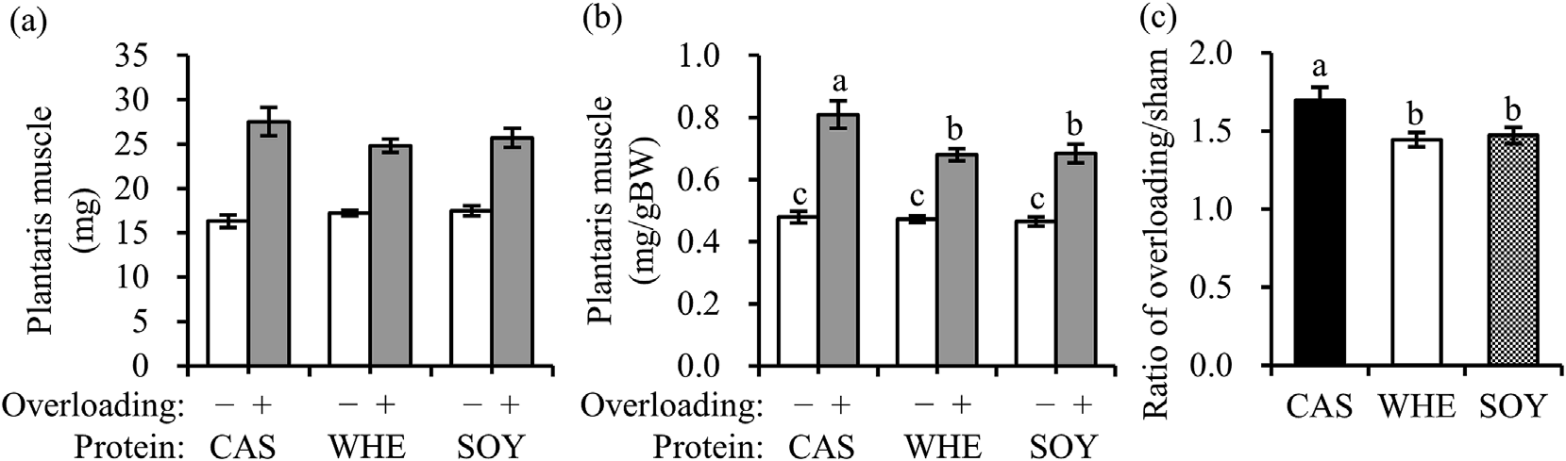
Effects of protein source on plantaris muscle weight and ratio of muscle hypertrophy. (a) Plantaris muscle wet weight, (b) relative plantaris muscle weight to body weight and (c) ratio of overload/sham-operated plantaris muscle weight. The values are expressed as means ± SEM (*n* = 10–11). Means without a common letter are significantly different (*p* < 0.05), using one-way ANOVA and Tukey for multiple comparisons. Caseinate; CAS, whey; WHE, soy; SOY.

Sham-operated plantaris muscle weight per body weight was similar in each group (Fig. 2a and b). In contrast, hypertrophy-induced plantaris muscle weight per body weight was significantly higher in CAS compared to that in WHE and SOY. The hypertrophy ratio of plantaris muscle was also significantly higher in CAS compared to that in WHE and SOY (Fig. 2c).

### 3.3 Other tissue weights

Liver, kidney, spleen, epididymal (epi) fat, and retroperitoneal and perirenal (ret) fat weight per body weight are shown in Table 3. Due to the difference in body weight gain, liver and spleen weights in SOY group were lower than in CAS and WHE groups. Epi fat and ret fat of SOY were higher than that of CAS and WHE, suggesting the increased body weight in SOY to be due to increase of fat.

**Table 3:** Tissue weights per body weight of caseinate, whey, and soy group.

	CAS	WHE	SOY
Liver	59.22 ± 0.99^a^	61.46 ± 1.14^a^	54.41 ± 1.49^b^
Kidney	16.88 ± 0.41	18.00 ± 0.42	17.20 ± 0.43
Spleen	2.98 ± 0.08^a^	3.08 ± 0.11^a^	2.58 ± 0.12^b^
Fat (epi)	21.83 ± 1.81^a^	23.15 ± 2.11^ab^	30.36 ± 2.78^b^
Fat (ret)	6.11 ± 0.57^a^	5.95 ± 0.53^a^	9.29 ± 1.14^b^
			(mg/g BW)

Tissue weights of CAS(caseinate), WHE(whey), and SOY(soy). The values are expressed as means ± SEM. Means without a common letter are significantly different (*p* < 0.05) using one-way ANOVA following Tukey for multiple comparisons. Epididymal; epi, retroperitoneal and perirenal; ret.

### 3.4 Locomotor activity

**Fig. 3:**
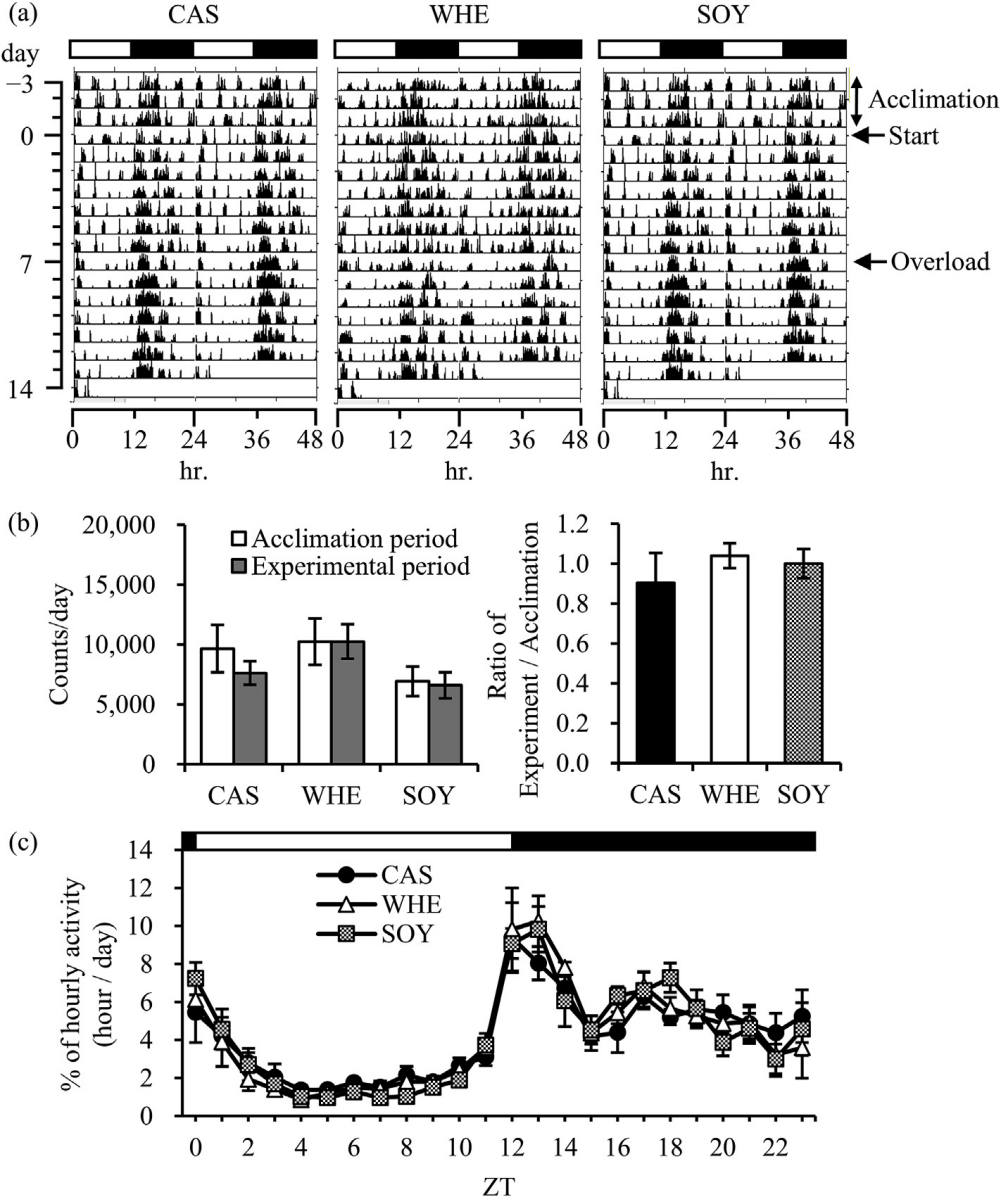
Effects of protein source on locomotor activity. Locomotor activity during experimental period. (a) Representative double-plotted actograms of locomotor activity determined by an area sensor in caseinate (CAS), whey (WHE), and soy (SOY) groups. White and balck bars mean light and dark periods, respectively. (b) Experimental period/acclimated period ratio and counts per day. The values are expressed as means ± SEM (*n* = 5–6). (c) Activity during day (ZT0-12) and night (ZT12-24) phase of CAS, WHE, and SOY (*n* = 5–6). Zeitgeber time; ZT.

To clarify the reasons for the differential body weight gain and muscle weight gain by the protein sources, locomotor activity of CAS, WHE, and SOY during the experiment was examined. Locomotor activities of each group, before and during the experimental period, are shown in Fig. 3. The average activity level over a 24-h period was not different across CAS, WHE, and SOY.

## 4 Discussion

In this study, we showed that the type of protein source (as in CAS, WHE, and SOY) affects functionally overloaded muscle hypertrophy, whereas there is no effect on sham-operated muscle weight. Caseinate induced higher muscle hypertrophy compared to whey and soy proteins, thus indicating that caseinate intake, as a daily protein source, may lead to muscle hypertrophy in a long-term intake/exercise model compared to whey and soy protein intake.

Leucine is reported to be important for muscle protein synthesis [21]. In this study, leucine and branched-chain amino acid (BCAA) content of whey was found to be approximately 1.2-fold higher than that of caseinate; however, muscle hypertrophy ratio was higher in CAS than in WHE. This result suggests that a slight difference of leucine and BCAA content may not be a determinant of hypertrophy in long-term intake experiment with an exercise model. In case of acute anabolic effect after exercise, a slight difference of leucine content did not affect muscle FSR rate (1–5 h) [22]. However, pre-sleep ingestion of caseinate increased overnight muscle protein synthesis rates [23]. The favorable effects of CAS may be due to its ability to maintain high levels of blood amino acids [7,8]; however, the relevant mechanism could not be demonstrated in this study.

Effect of protein source on muscle hypertrophy may vary depending on exercise intensity as well. For example, Hashimoto et al. had reported the soy protein supplementation increased skeletal muscle volume compared with caseinate supplementation in the subjects under the low physical activity but not high physical activity [24]. Additionally, in the bedridden patients, caseinate supplementation significantly increased skeletal muscle volume than no supplementation while its significant increase was not observed by soy protein supplementation [24]. Therefore, the effect of protein source on muscle hypertrophy may depend on exercise intensity.

In this study, SOY group showed increased fat mass and body weight as food intake increased. The reason behind this increased food consumption is not clear; there was no difference in the amount of locomotor activity as well. Contrary to our results, Ijaz et al. had reported that consumption of caseinate diet is increased compared to that of soy and beef, although it did not affect body weight gain [25]. Therefore, food consumption may vary across the experimental conditions. The effect of soy protein on weight gain has been reported earlier [26]. In experiments with high-fat diet-fed rats, no difference in food intake and body weight was seen between whey protein-fed group and soy protein-fed group [27]. In another rat-related experiment, increase of body weight in the soy-fed group was suppressed and there was no difference in intake amount across the whey, caseinate, and soy protein-fed groups [28]. A previous report had suggested that soy protein suppressed high-fat induced weight gain, without affecting food intake, and its effect was abolished in germ-free mice [29]. Such findings suggest that both experimental conditions and sources of protein may influence the soy protein effect on body weight. In our experiment, sham-operated plantaris muscle weights, without body weight correction, did not change across the groups, thus suggesting that the differential body weight gain cannot affect hypertrophy results.

In the present study, we showed that caseinate intake induces higher muscle hypertrophy compared to whey and soy protein intake, in a long-term ingestion with exercise-mimicking hypertrophy model. Though the underlying reason for different protein sources causing different levels of muscle hypertrophy has not been clarified yet, our results suggest caseinate to be more effective for muscle hypertrophy than whey and soy proteins.

## Funding

This work was supported by the Council for Science, Technology, and Innovation, SIP, “Technologies for creating next-generation agriculture, forestry, and fisheries” (funding agency: Bio-oriented Technology Research Advancement Institution, NARO) (S.S.) and Japan Society for the Promotion of Science (JSPS) KAKENHI Grant Number 17K18176 (S.A.).

## Authors’ contributions

A.S. and S.S. designed the study. A.S., R.H. and T.S. carried out experiments. A.S. analyzed the data. A.S. and S.S. wrote the manuscript. All authors have read and approved the manuscript.

## Conflicts of interest

The authors declare that the research was conducted in the absence of any commercial or financial relationships that could be construed as a potential conflict of interest.
