# Supplementary material for: Effect of different sources of dietary protein on muscle hypertrophy in functionally overloaded mice
Source: Biochem Biophys Rep. 2019 Sep 10;20:100686. doi: 10.1016/j.bbrep.2019.100686 (PMC6742840; doi:10.1016/j.bbrep.2019.100686)
Supplement: Multimedia component 1 [file mmc1.docx]

Supplementary Table 1 Composition of diets

| Ingredient (g) | CAS | WHE | SOY |
| --- | --- | --- | --- |
| Calcium caseinate | 200 | 0 | 0 |
| Whey protein concentrate | 0 | 238 | 0 |
| Isolated soy protein | 0 | 0 | 232 |
| Cysteine | 3 | 3 | 3 |
| Corn starch | 532 | 494 | 500 |
| Sucrose | 100 | 100 | 100 |
| Cellulose | 50 | 50 | 50 |
| Soybean oil | 70 | 70 | 70 |
| Mineral mix | 35 | 35 | 35 |
| Vitamin mix | 10 | 10 | 10 |
| Total | 1000 | 1000 | 1000 |

CAS, caseinate; WHE, whey protein concentrate; SOY, isolated soy protein
